# Mechanics of the foot and ankle joints during running using a multi-segment foot model compared with a single-segment model

**DOI:** 10.1371/journal.pone.0294691

**Published:** 2024-02-13

**Authors:** Justin C. Wager, John H. Challis

**Affiliations:** 1 Department of Physical Therapy and Human Movement Science, Sacred Heart University, Fairfield, Connecticut, United States of America; 2 Biomechanics Laboratory, Pennsylvania State University, University Park, Pennsylvania, United States of America; Ningbo University, CHINA

## Abstract

The primary purpose of this study was to compare the ankle joint mechanics, during the stance phase of running, computed with a multi-segment foot model (MULTI; three segments) with a traditional single segment foot model (SINGLE). Traditional ankle joint models define all bones between the ankle and metatarsophalangeal joints as a single rigid segment (SINGLE). However, this contrasts with the more complex structure and mobility of the human foot, recent studies of walking using more multiple-segment models of the human foot have highlighted the errors arising in ankle kinematics and kinetics by using an oversimplified model of the foot. This study sought to compare whether ankle joint kinematics and kinetics during running are similar when using a single segment foot model (SINGLE) and a multi-segment foot model (MULTI). Seven participants ran at 3.1 m/s while the positions of markers on the shank and foot were tracked and ground reaction forces were measured. Ankle joint kinematics, resultant joint moments, joint work, and instantaneous joint power were determined using both the SINGLE and MULTI models. Differences between the two models across the entire stance phase were tested using statistical parametric mapping. During the stance phase, MULTI produced ankle joint angles that were typically closer to neutral and angular velocities that were reduced compared with SINGLE. Instantaneous joint power (p<0.001) and joint work (p<0.001) during late stance were also reduced in MULTI compared with SINGLE demonstrating the importance of foot model topology in analyses of the ankle joint during running. This study has highlighted that considering the foot as a rigid segment from ankle to MTP joint produces poor estimates of the ankle joint kinematics and kinetics, which has important implications for understanding the role of the ankle joint in running.

## Introduction

Joint power analyses have suggested that the ankle is the largest contributor to lower limb positive work and a primary generator of power during the stance phase of running [e.g., 1,2]. However, traditional lower limb biomechanical models treat the foot as a single rigid body which may incorrectly measure foot motion and, therefore, ankle motion [3,4].

The influence of foot model topology on ankle kinematics and kinetics has not been explored for running; the kinematic and kinetics of the ankle joint during running computed not accounting for the joints distal to the ankle joint have not been compared with those from a more detailed model of the foot which accounts for those joints. Such an investigation is warranted to bolster our understanding of lower limb mechanics during running.

Recent analyses of walking have used foot models that better capture the foot’s behavior. Siegel et al. (1996) analyzed foot segment energy during walking using a deformable model that included energy changes due to movement of the foot’s center of mass. This model demonstrated improved agreement with the work-energy theorem and has been used to characterize the energy profile of the combined foot-ankle system [5–7]. In addition, multi-segment foot models (typically modeling the hindfoot, forefoot, and hallux separately) have gained traction in clinical investigations [8,9]. Multi-segment foot models produce, for walking, different ankle kinematics and kinetics compared with a single segment foot model. For example, the ankle extension angles and angular velocities are decreased [10], and ankle joint power during the stance phase is typically lower when using a multi-segment foot model compared with a single segment foot model [11]. While multi-segment foot models have improved our understanding of foot and ankle function during walking, most studies of running continue to use a single segment foot model. Therefore, ankle joint power values and the distribution of power among the lower limb joints during running warrants reinvestigation using a multi-segment foot model. Furthermore, the arch of the foot may play an important role during running [12–14] and estimating the mechanical energetics of the foot joints may shed additional light on the role of the foot during running.

A common approach for the analysis of running has been to model the foot at a single segment defined from the ankle to the metatarsophalangeal joint [e.g., 15–17]. Given the evidence that ankle joint kinematics and kinetics can be overestimated for walking when the foot is modeled as single segment [8–11] and given the greater ranges of motion which can occur during running compared with walking [18] a single segment model of the foot may be less appropriate for running. There have been studies showing that during running there are angle changes of the joints of the foot [e.g., 19,20]. These angle changes mean when the foot is modeled as a single segment some of the foot joint angle changes are subsumed into the ankle joint kinematics. Inverse dynamics requires knowledge of joint kinematics, and with a Newton-Euler approach errors in the moments estimated for one joint are propagated to other joints in the system. Therefore, if ankle angles are incorrect this will therefore directly influence computed ankle moments, and will impact the moments computed for other joints in the system. While there have been studies of running using more detailed models of the foot [e.g., 19,20], to date there have been no studies which have compared ankle kinematics and kinetics during running for single and multiple segment foot models to establish any differences.

During the push-off phase of running ankle joint positive power constitutes the majority of the mechanical power generated by the lower limb during stance [16,21]. Analysis of the moments and power at the ankle joint are important in of themselves, but are also important in understanding the behavior of the triceps surae muscles. For example, ankle kinematics have been used to estimate Achilles tendon load during running [22], to estimate the actions of the triceps surae muscles [23], and to examine the loads at potential injury sites [24]. All of these analyses rely on accurate estimates of ankle joint kinematics.

The primary purpose of this study was to compare the ankle joint mechanics, during the stance phase of running, computed with a multi-segment foot model (MULTI; three segments) with a traditional single segment foot model (SINGLE). Based on evidence from walking studies [10,11] it was hypothesized that MULTI compared with SINGLE would produce reduced values for ankle joint kinematics and kinetics. The secondary hypothesis was that for the stance phase of running the work performed at the ankle joint computed from SINGLE, due the work-energy theorem, would be distributed across the ankle and midfoot joints for MULTI, where the midfoot joint is defined as a collective representation of the midtarsal and tarsometatarsal joints.

## Methods

### Participants

Seven heel-striking runners with no musculoskeletal injuries in the past six months were recruited for this study. Based on pilot data an *a priori* power analyses indicated that for the analyses of time series data (e.g., joint kinematics) using statistical parametric mapping, and for traditional statistical analysis of non-time-based scalar data (e.g., joint work) that at least six subjects were required for statistical power to exceed 80% with a large effect size [25,26]. All subjects provided voluntary written informed consent for the study whose protocols were approved by the Institutional Review Board (Study# 00007075). Data collection was conducted 2017–18, and data analyses commenced in 2019. All subject data was anonymous.

### Data collection

**Fig 1:**
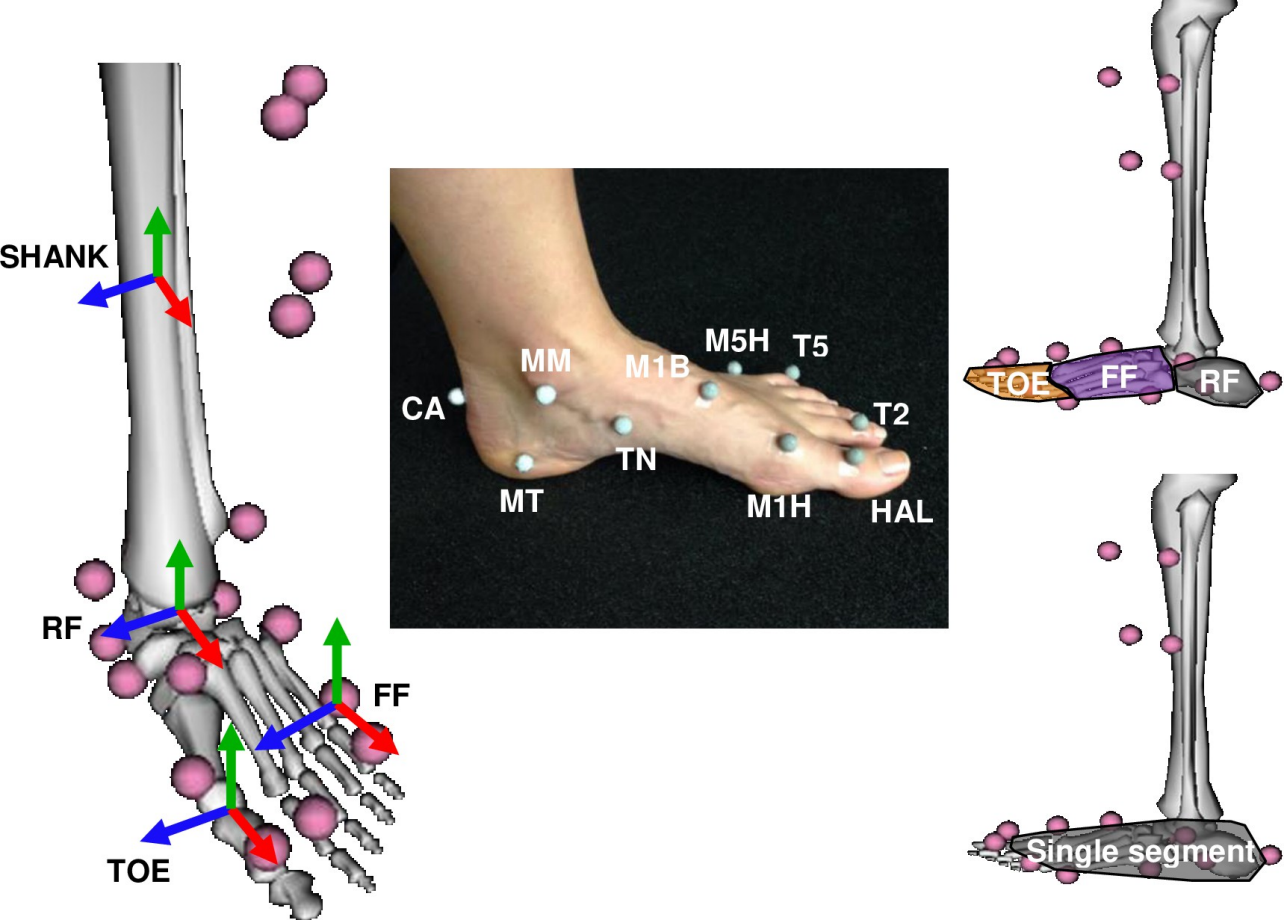
The segment reference frames for the multi-segment foot model (left), marker set (center), and the two foot models (right). The SHANK reference frame was the same for both models and the single segment foot reference frame in SINGLE was the same as the RF reference frame in MULTI. The multi-segment foot model included three segments (RF = rearfoot, FF = forefoot, and TOE). In each reference frame, the red arrow indicates the x-axis, the green arrow indicates the y-axis, and the blue arrow indicates the z-axis. The labelled markers are those of the: M1B - base of the first metatarsal; M5H - fifth metatarsal head; T5—distal edge of middle phalanx of the fifth toe; M1H - first metatarsal head; and HAL—distal edge of the proximal phalanx of the hallux.

Each subject ran barefoot down a 15m runway at 3.1 m/s. Passive retroreflective skin markers were placed on bony landmarks of the lower leg (Fig 1). Marker positions were tracked at 150 Hz by a seven-camera motion capture system (Motion Analysis Corporation, Mountain View, CA). Ground reaction forces (GRF) and moments were collected at 1500 Hz from a 90 x 60 cm force plate (Model 9287A, Kistler Instrument Corporation, Amherst, NY). All data were lowpass filtered in the forward and reverse directions using a second-order recursive Butterworth filter with a 10 Hz (marker positions) or 45 Hz (GRF) cutoff frequency selected by analysis of residuals between filtered and unfiltered signals [27]. While some authors have recommended using the same cut-off for kinematic and force plate data [28], although there is no consensus [29], here to avoid attenuation of the GRF signal a higher cut-off was used for these data.

Inertial properties and center of mass (COM) locations for the shank were calculated from the subject mass and limb dimensions [30]. The foot was divided into three separate segments (rearfoot, forefoot, and toes). For these segments the masses and moments of inertia were based on [31], which required splitting their hindfoot values into equal halves and assigning the resulting values to the rearfoot and forefoot segments. Foot segment center of mass (COM) locations were calculated as the geometric average of each segment’s marker locations. The origin of each segment reference frame was located at the segment’s COM.

### Data processing

Segment positions and orientations were computed from the marker locations [32], with orientations expressed relative to the neutral standing position (with the foot unweighted, similar to Caravaggi et al. [33]) and decomposed into Cardan angles using a ZXY sequence. Joints defined were the ankle joint (RF relative to shank), the midtarsal joint (FF relative to RF), and the MTP joint (TOE relative to FF). Rotations about the x-axis corresponded to frontal plane motion (inversion/eversion), about the y-axis corresponded to transverse plane motion (ab/adduction), and about the z-axis corresponded to sagittal plane motion (flexion/extension).

Ankle angles were determined similarly for both SINGLE and MULTI, but the foot model differed (Fig 1). SINGLE used one rigid segment to represent all the bones between the ankle and metatarsophalangeal (MTP) joints, with the toes neglected except as a point at which the ground reaction forces could act. The ankle was defined as the joint between this single foot segment and the shank. MULTI adopted a three segment foot model and definitions of joint angles of a previously validated model [34]. MULTI comprised three segments: 1) rearfoot (RF): calcaneus; 2) forefoot (FF): all bones between the navicular and metatarsals, inclusive; and 3) toes (TOE): all five toes lumped into one segment [34]. The ankle joint in MULTI was defined as the articulation between the shank and RF segment. Reference frame definitions for the single segment of SINGLE and the RF segment of MULTI were identical to allow direct comparison of the ankle kinematics between the two models (Table 1).

**Table 1:** Definitions of unit vectors used to define segment reference frames for the segments of the multi-segment foot model. The single segment foot model shares the same reference frame definition as the rearfoot to standardize the ankle joint convention. See Fig 1 for marker locations.

Segment	x-axis (inv/eversion)	y-axis (ab/adduction)	z-axis (plantar/dorsiflexion)
**Rearfoot**	CA to M1H	Cross product: *x*-axis and vector from CA to midpoint of M1H and M5H	Cross product: *x* and *y* axes
**Forefoot**	Cross product: *y* and *z* axes	Cross product of *z*-axis and vector from M5H to M1H	M1H to M5H
**Toes**	M1H to HAL	Cross product: *x*-axis and vector from T5 to HAL	Cross product: *x* and *y* axes

***Note***: CA—insertion of the Achilles tendon on the calcaneus; M1H - first metatarsal head; M5H - fifth metatarsal head; T5—distal edge of middle phalanx of the fifth toe; HAL—distal edge of the proximal phalanx of the hallux.

Resultant joint forces and moments during stance were determined through recursive Newton-Euler inverse dynamics (using custom-written MATLAB code). The ankle joint center was the midpoint between markers placed on the lateral malleolus and the medial malleolus for SINGLE and MULTI. For MULTI two additional joint centers were defined. The midtarsal joint center was the midpoint between markers on the navicular tuberosity and base of the fifth metatarsal. The MTP joint center was at the location of the head of the first metatarsal marker. At each joint and for each timepoint during the stance phase, the instantaneous mechanical power, *P_j_*, was calculated as the scalar product of the resultant joint moment and joint angular velocity. Instantaneous power during stance was integrated to obtain the positive, negative, and net-work at each joint. Resultant joint moments, powers, and work were normalized to body mass.

Performing inverse dynamics with MULTI required estimations of the forces and moments acting on each foot segment (RF, FF, TOES), however all of these segments could be in contact with the ground with the GRF distributed among these segments. Two GRF decomposition techniques were tested during a pilot study: 1) the subject was asked to perform targeted landings on two adjacent force plates with each plate measuring the forces on different portions of the foot, and 2) the entire GRF was assigned to only one-foot segment at a time, with the GRF assigned to the segment whose boundary (defined by the proximal and distal joint centers) contained the center of pressure (COP) position. Sensitivity analyses were performed to determine the precision of the estimated resultant joint moments determined using both techniques [35]. The two techniques produced resultant joint moments which differed by amounts smaller than the precision with which these moments can be measured. As the two techniques gave the same results and the first technique required force plate targeting the second approach was used.

### Statistical analysis

Statistical analyses for time series data were performed using statistical parametric mapping (SPM), which tests for differences in vector fields instead of scalar values and are more appropriate for time series data that do not vary randomly [36,37]. Three-dimensional data (i.e., the time series of each of the ankle joint angles), were tested with SPM paired Hotelling’s T^2^ tests. If significance was reached (*α* < 0.05), SPM paired t-tests between SINGLE and MULTI were performed on the three vector components using a Sidak corrected threshold of *α* = 0.0170. For 1-dimensional data (e.g., instantaneous joint power), SPM paired t-tests were performed between SINGLE and MULTI. For non-time-based scalar data (e.g., joint work), traditional paired Student’s t-tests were performed with Bonferroni correction. Normality of data was confirmed prior to statistical analysis [38]. Statistical tests were performed in Python 3.6.1 (Anaconda Distribution, Continuum Analytics, Austin, TX) with the spm1d package (version M.0.4.5, www.spm1d.org) [39] or MATLAB R2016a (version 9.0.0.361340).

## Results

### Ankle kinematics comparisons

**Fig 2:**
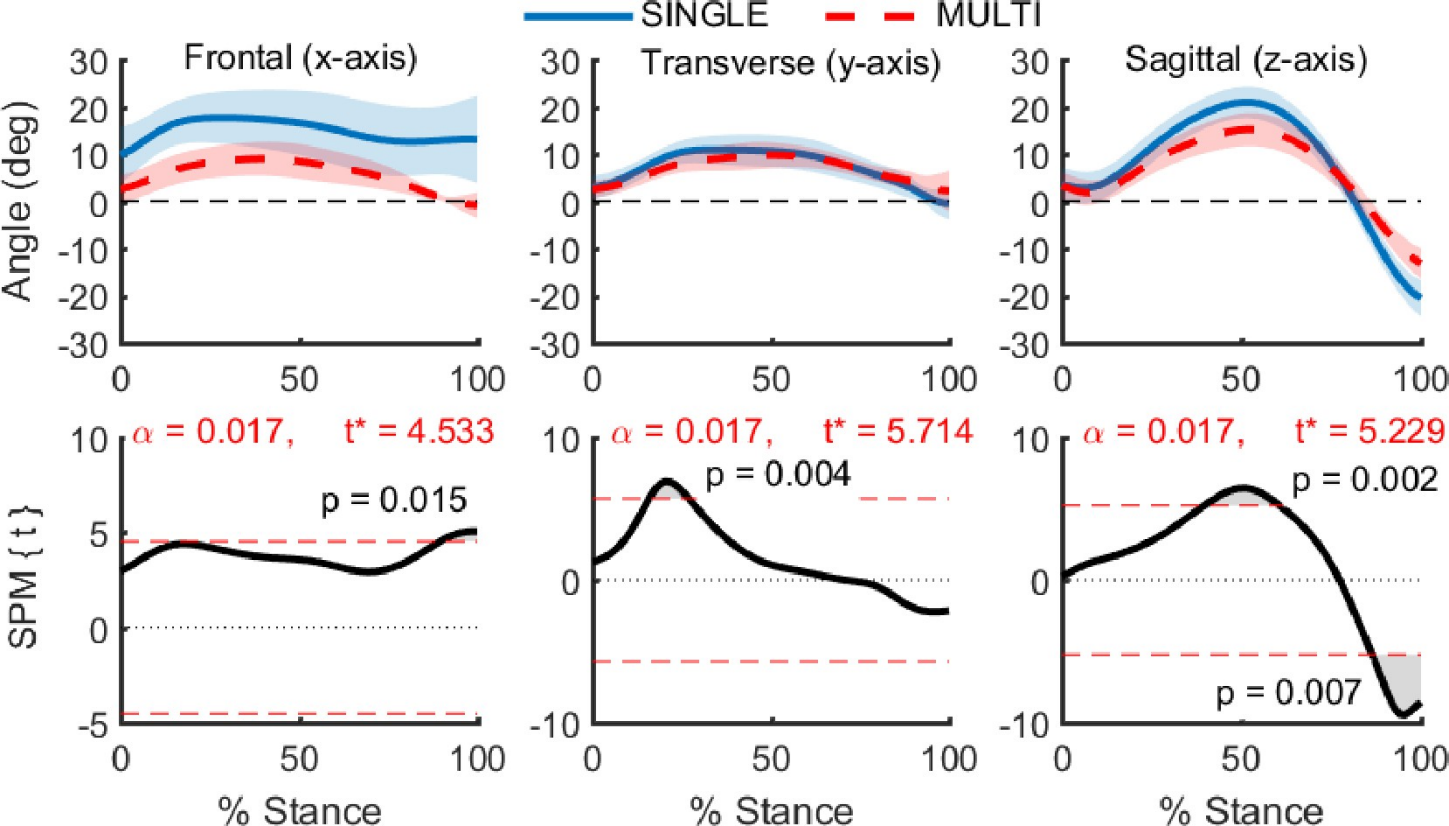
**Comparison of ankle angles during stance computed using two different foot models: SINGLE—single segment foot (solid blue), MULTI—multi-segment foot (dotted red).** Data computed from barefoot running at 3.1 m/s. Shaded regions show ± 1 S.D. The top row shows ankle angles throughout the stance phase. Angle conventions are inversion(+)/eversion(-), abduction(+)/adduction(-), and dorsiflexion(+)/plantarflexion(-). The bottom row shows results from paired t-tests btween SINGLE and MULTI, with the red dotted lines representing the t-statistic threshold for statistical significance. Gray shaded areas outside of the red dotted lines show regions with significant differences between SINGLE and MULTI.

There were significant differences in ankle angle between SINGLE and MULTI. *Post-hoc* paired t-tests on the three ankle angles revealed significant differences between MULTI and SINGLE (Fig 2). During early stance (16–27%), MULTI displayed less ankle adduction than SINGLE. During midstance, MULTI displayed reduced dorsiflexion from 40–61% of stance. During late stance, MULTI exhibited both reduced plantarflexion (86–100% stance) and reduced adduction (90–100% stance) angles compared with SINGLE.

**Fig 3:**
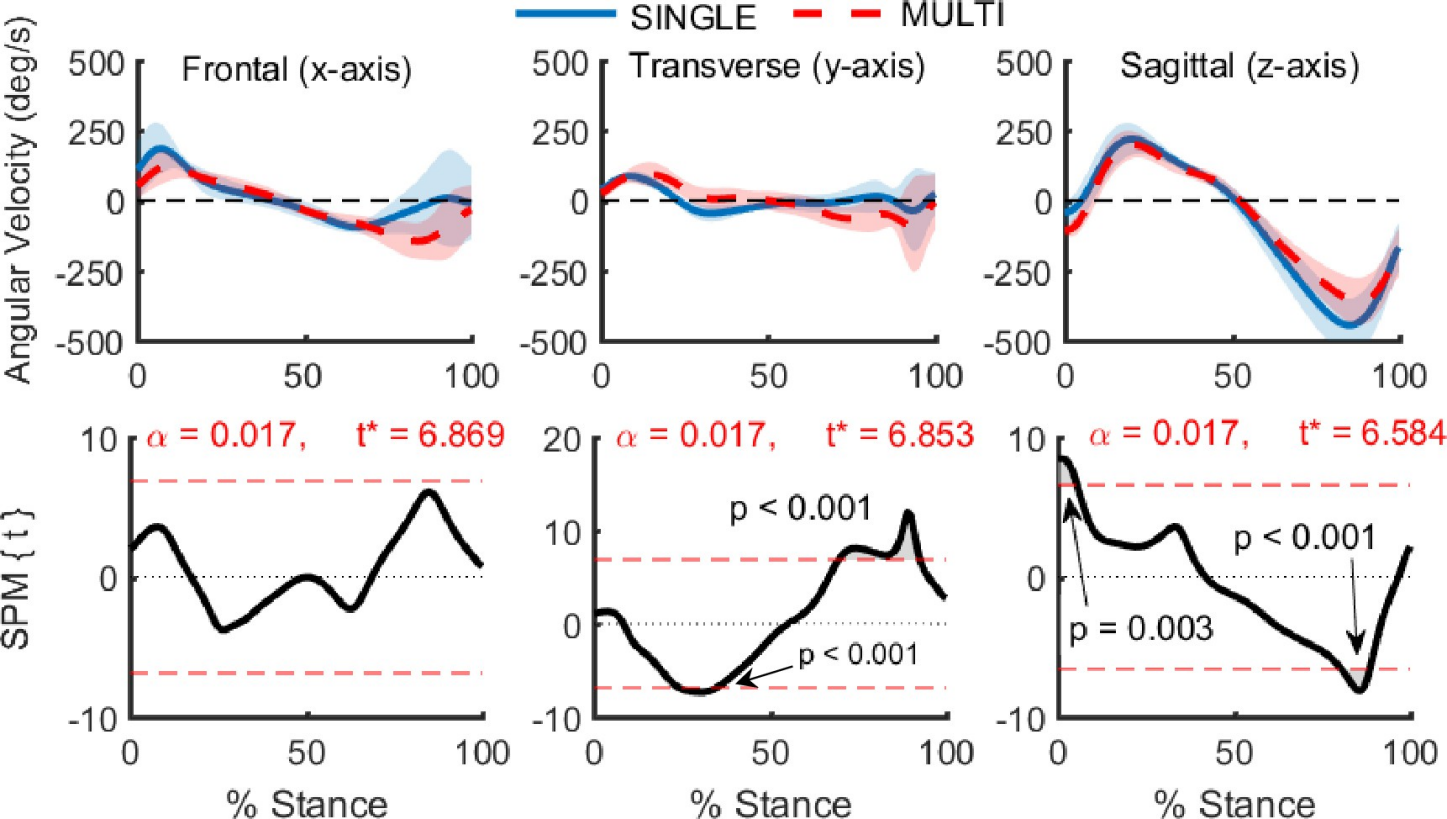
**Comparison of ankle angular velocities during stance computed using two different foot models: SINGLE—single segment foot (solid blue), MULTI—multi-segment foot (dotted red).** Data computed from barefoot running at 3.1 m/s. Shaded regions show ± 1 S.D. The top row shows ankle angular velocities throughout the stance phase. Angle conventions are inversion(+)/eversion(-), abduction(+)/adduction(-), and dorsiflexion(+)/plantarflexion(-). The bottom row shows results from paired t-tests btween SINGLE and MULTI with the red dotted lines representing the t-statistic threshold for statistical significance. Gray shaded areas outside of the red dotted lines show regions with significant differences between SINGLE and MULTI.

Ankle angular velocity was significantly different between SINGLE and MULTI. *Post-hoc* paired t-tests on the angular velocity components between SINGLE and MULTI revealed differences about the ab/adduction and dorsi-/plantar-flexion axes (Fig 3). MULTI exhibited reduced dorsiflexion velocity during early stance and reduced plantarflexion velocity during late stance. Ab/adduction angular velocities of the two models occasionally differed in sign (Fig 3, top center plot). Near 30% of stance, MULTI displayed little to no ankle rotation in the direction of abduction, while SINGLE displayed a small rotation velocity towards adduction. The opposite pattern was found during late stance with MULTI reporting rotations in the adduction direction and SINGLE reporting close to zero angular velocity.

### Ankle kinetics comparisons

Ankle resultant joint moments computed using the two models were significantly different. *Post-hoc* paired t-tests on the joint moment components between SINGLE and MULTI displayed significant differences about the inversion/eversion and dorsi-/plantar-flexion during early and midstance, and for all three axes during late stance. These differences were statistically significant but relatively small in magnitude so likely of little functional significance.

**Fig 4:**
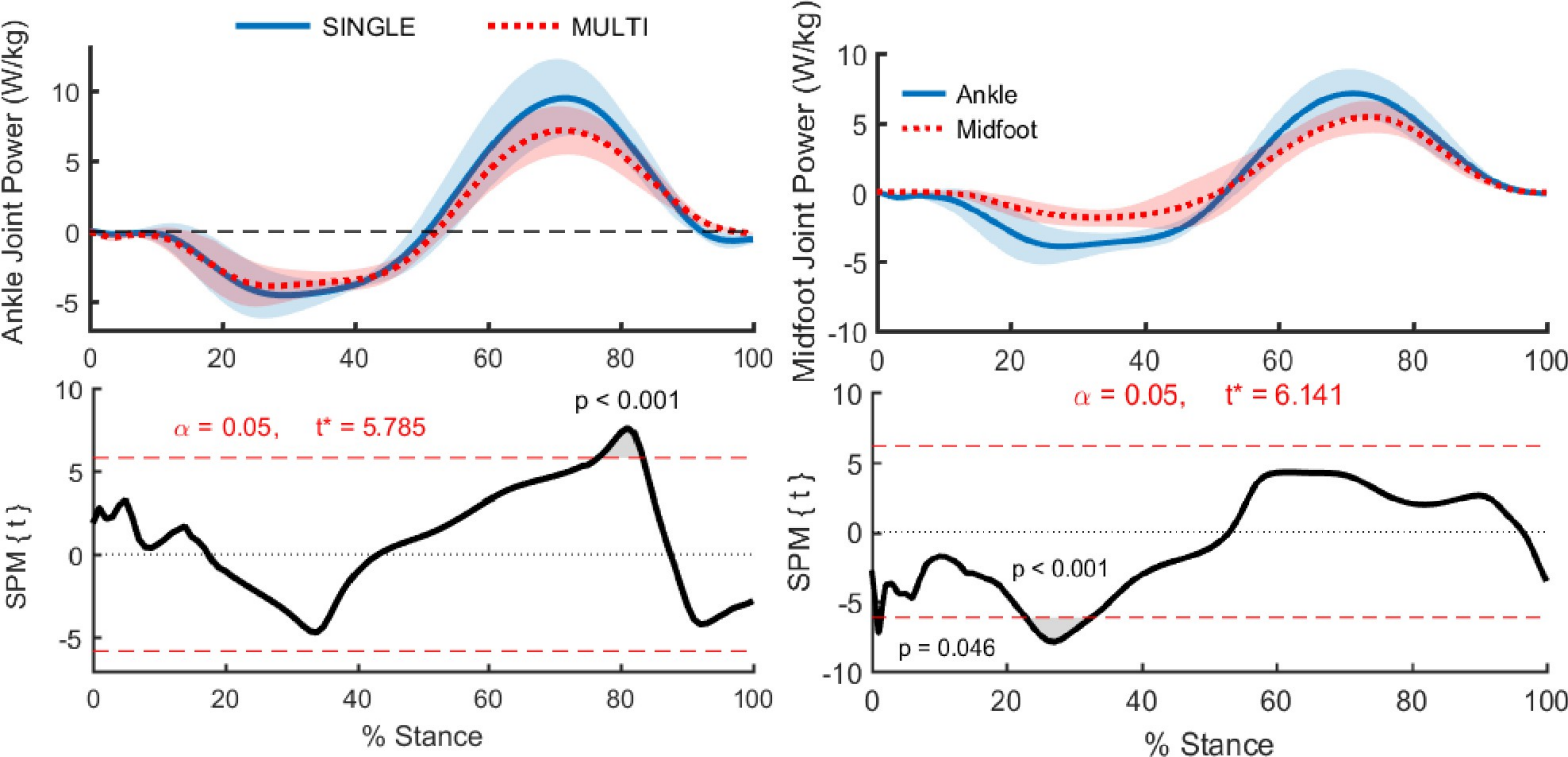
Comparison of instantaneous ankle and midfoot joint power during stance computed using two different foot models. The foot models were: SINGLE—single segment foot (solid blue), and MULTI—multi-segment foot (dotted red). The "midfoot joint” is defined as the orientation of the FF segment relative to the RF segment and represents the collective function of all the joints between the talus and metarasals (midtarsal and tarsometatarsal joints). For both A and B: data computed from barefoot running at 3.1 m/s. Shaded regions show ± 1 S.D. Bottom plot shows results from a paired t-test, with the red dotted lines representing the t-statistic threshold for statistical significance. Gray shaded areas outside of the red dotted lines show regions with significant differences between SINGLE and MULTI (A) or the ankle and midfoot joints (B).

Instantaneous ankle joint power was significantly different between SINGLE and MULTI during 76–83% of stance (*p <* 0.001; Fig 4A). During this period, ankle power computed with MULTI was lower than the ankle power computed with SINGLE with a mean difference between the models (across all subjects) ranging from 0.9 to 3.1 W∙kg^-1^. This represents a difference of 18–22% of the ankle power computed by SINGLE and suggests that traditional single segment foot models overestimate ankle joint power during a portion of push-off. These joint power differences were primarily due to the differences in joint angular velocity between the models, as these differences were much larger than the joint moment differences.

**Fig 5:**
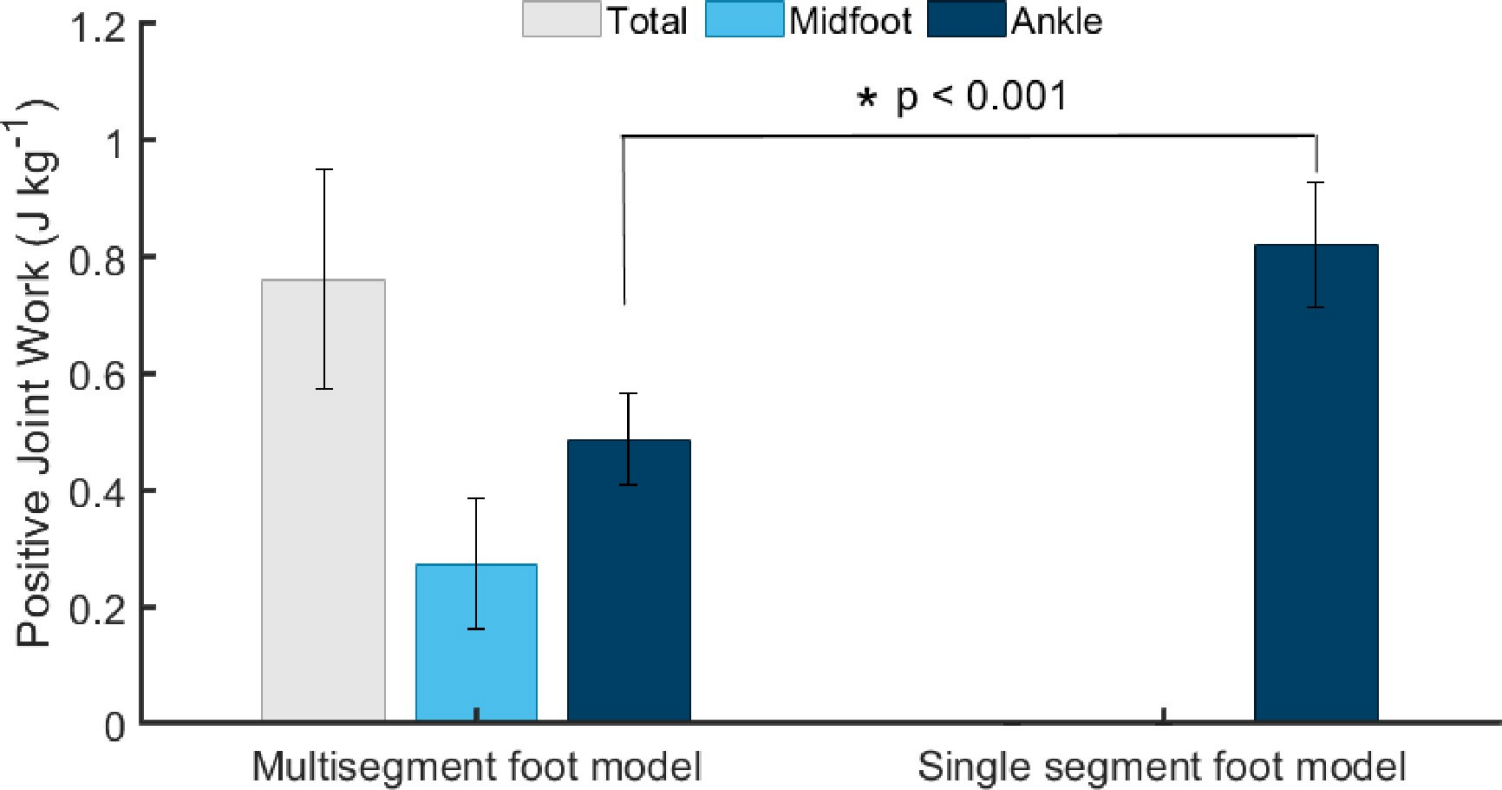
Comparison of positive joint work performed at each joint in the two foot models. The multi-segment foot model (MULTI) is the left bar group, and the single segment foot model (SINGLE) is the right bar. Ankle positive work (dark blue) was significantly different between the foot models, but the summed ankle and midfoot from MULTI (gray bar) was similar to ankle joint work from SINGLE (dark blue), suggesting that SINGLE captured midfoot joint power in the ankle joint power.

Positive ankle joint work was significantly different between models, with MULTI generating 41% less positive work than SINGLE during push-off (Fig 5; mean difference = 0.33 ± 0.03 J∙kg^-1^; *p <* 0.001). A significant difference in negative ankle joint work was also detected between models, with MULTI absorbing 28% less energy during early stance (mean difference = 0.12 ± 0.06 J∙kg^-1^; *p =* 0.001).

### Midfoot joint mechanics

MULTI revealed that during stance, the midfoot joint absorbed energy during early stance and generated energy during late stance, similar to the temporal pattern found at the ankle (Fig 4B). A SPM paired t-test between the ankle and midfoot joint powers revealed that the ankle absorbed more power than the midfoot during portions of early stance (at foot contact, *p* < 0.046, and at 23–33% of stance, *p* < 0.001). However, the ankle and midfoot joint generated statistically similar amounts of power throughout late stance. Despite similar power generation, positive joint work during stance was greater for the ankle than the midfoot joint (*p* = 0.01; difference = 0.25 ± 0.17 J·kg^-1^; Fig 5). The sum of the positive work performed at the midfoot joint and ankle joint in MULTI was similar to the positive work performed at the ankle joint in SINGLE. This suggests that positive ankle joint work in SINGLE was a combination of work done by the joints of the midfoot and the ankle joint combined, thereby overestimating the ankle’s contribution to positive work during stance.

## Discussion

The primary purpose of this study was to compare the ankle joint mechanics, during the stance phase of running, computed with a multi-segment foot model (MULTI; three segments) with a traditional single segment foot model (SINGLE). This study has demonstrated that foot model topology can influence the analysis of ankle joint mechanics during running. It was hypothesized that a multi-segment foot model (MULTI) would provide reduced ankle joint kinematics and kinetics values compared with a single segment foot model (SINGLE). This hypothesis was supported as stance phase ankle joint angles, angular velocities, joint moments, joint power, and positive joint work were lower for MULTI compared with SINGLE. As joint kinematics and kinetics play a central role in our interpretation of joint and muscle function during locomotion, these results provide evidence that oversimplification of the foot in biomechanical modeling can produce potentially incorrect outcomes for ankle joint mechanics. The secondary hypothesis was that for the stance phase of running the work performed at the ankle joint from SINGLE would be distributed across the ankle and midfoot joints for the MULTI model. This hypothesis was supported as the stance phase ankle positive work from SINGLE was equal to the sum of the positive work from the midfoot and ankle joint (Fig 5). This study therefore suggests that single segment foot models omit potentially important information about energy contributions from the arch of the foot during running, with the midfoot joint representing the arch of the foot. In MULTI, the midfoot joint generated substantial energy during stance. These results indicate that the arch of the foot plays a potentially important energetic role during running, as it both absorbs energy during early stance and generates energy during late stance. Ignoring midfoot joint work by using models that are too simple can incorrectly attribute work done by the muscles, tendons, and ligaments of the foot to the muscles and tendons of the ankle joint.

The work and power differences between the two models were caused mainly by kinematic differences. Ankle joint angles between SINGLE and MULTI were different across all three axes of motion, with the largest differences occurring for the ab/adduction and dorsi-/plantar-flexion axes. Overall, temporal profiles were similar, but ankle angles from MULTI tended to remain closer to neutral than those from SINGLE. For ankle dorsi-/plantar-flexion MULTI exhibited 28.5 degrees of total excursion while SINGLE exhibited 41.7 degrees of total excursion. The lower total excursion of MULTI was due to both a reduction in dorsiflexion during midstance and a reduction in plantarflexion during late stance (Fig 2). The excursion from MULTI was close to that reported by the bone pin investigations [19].

The differences in angular velocity were the main cause of the differences in ankle joint power, as the differences in ankle joint moments were small and occurred only during early and terminal stance (88–100% of stance). During the periods where the foot models produced different ankle joint moments, ankle joint power was not significantly different. In SINGLE, the foot (ankle to MTP joints) was tracked by three markers distributed along the length of the foot (posterior calcaneus, 1^st^ and 5^th^ metatarsal heads). Therefore, this model captured midfoot joint motion as ankle joint motion and increased the ankle joint excursion and angular velocity during stance, particularly during push-off. Experimental ankle kinematics are widely used to validate musculoskeletal models and tune model parameters [40], but these results suggest caution when the experimental kinematics are from a single segment foot model.

Throughout stance, the midfoot joint generated a plantarflexion moment, with a peak of 1.95 ± 0.25 N·m·kg^-1^. This resulted in substantial midfoot joint power during push-off as the arch shortened, which was not statistically different from ankle joint power (Fig 4B). The biological source of this mechanical power was likely in part the passive tissues that cross the midtarsal and tarsometatarsal joints. Energy transfer between the MTP joints and the arch during running is thought to occur via the plantar aponeurosis [14,41] and could also occur via the extrinsic toe flexors (FDL and FHL), which cross the ankle joint and function isometrically during walking [42].

The kinematics and kinetics of running estimated using the SINGLE foot model produced results qualitatively similar to those previously reported using the same foot model [16]. The kinematics estimated using MULTIPLE produced kinematics qualitatively similar to those from a previously reported study using a similar foot model [43,44], while the kinetics estimated were qualitatively similar to those previously reported using a similar foot model [e.g., 44, 45]. These comparisons provide evidence that the kinematics and kinetics reported here for either foot model are representative of those obtained in earlier studies, and highlights the importance of employing a foot model of sufficient complexity to accurately capture foot kinematics and kinetics and therefore ankle joint kinematics and kinetics.

Limitations to this study were related to both the modeling and experimental methodology. Modeling the foot as a three-segment system required assumptions about the mobility of the foot during locomotion. In MUTLI, ankle rotations were defined as rotation of the calcaneus relative to the tibia, which therefore includes both talocrural joint and subtalar joint rotations. During the stance phase of running, rotations of the calcaneus relative to the talus are 5–6 degrees in the sagittal and transverse planes, and 8 degrees in the frontal plane [19]. Though this may have influenced the estimated ankle joint power, the muscles that control the talocrural joint also partially control the subtalar joint, and many consider the “ankle joint” to be both of these joints. SINGLE also captured the same subtalar joint rotations within ankle joint rotation, therefore, this should not affect the comparisons between the models. In MULTI, the forefoot segment comprised all bones between the talus and phalanges and therefore represented the collective motion of all joints within that range. This definition of the forefoot segment has been shown to violate the rigid body assumption [34]. While this produced error mostly outside the sagittal plane, segment deformation could nonetheless influence the midfoot joint power during late stance. A further violation of the rigid body assumptions arises due the deformation at the soft-tissue structures of the foot (e.g., the heel pad), the integration of standard kinematic data collection methods in biomechanics with sophisticated finite-element of models of the human foot [e.g., 46] will help address this shortcoming.

In conclusion, this study has provided evidence that ankle joint kinematics and kinetics during running differ as a function of foot model topology. This study has highlighted that considering the foot as a rigid segment from ankle to MTP joint produces poor estimates of the ankle joint kinematics and kinetics, which has important implications for understanding the role of the ankle joint in running. The use of a multi-segment foot model, as opposed to the traditional single segment foot model, resulted in altered kinematics characterized by a more neutral position of the ankle, reduced sagittal plane angular velocity throughout stance, and increased transverse plane velocity during push-off. Ankle joint power and positive work during late stance were reduced by approximately 25% when using a multi-segment foot model compared with a single segment foot model. The multi-segment foot model also revealed that the arch of the foot may play an important energetic role during stance, as midfoot joint power generation during late stance was not significantly different from ankle joint power generation. These results show the importance of tracking the calcaneus separately from the rest of the foot and including a midfoot joint in studies of the human ankle during running.
